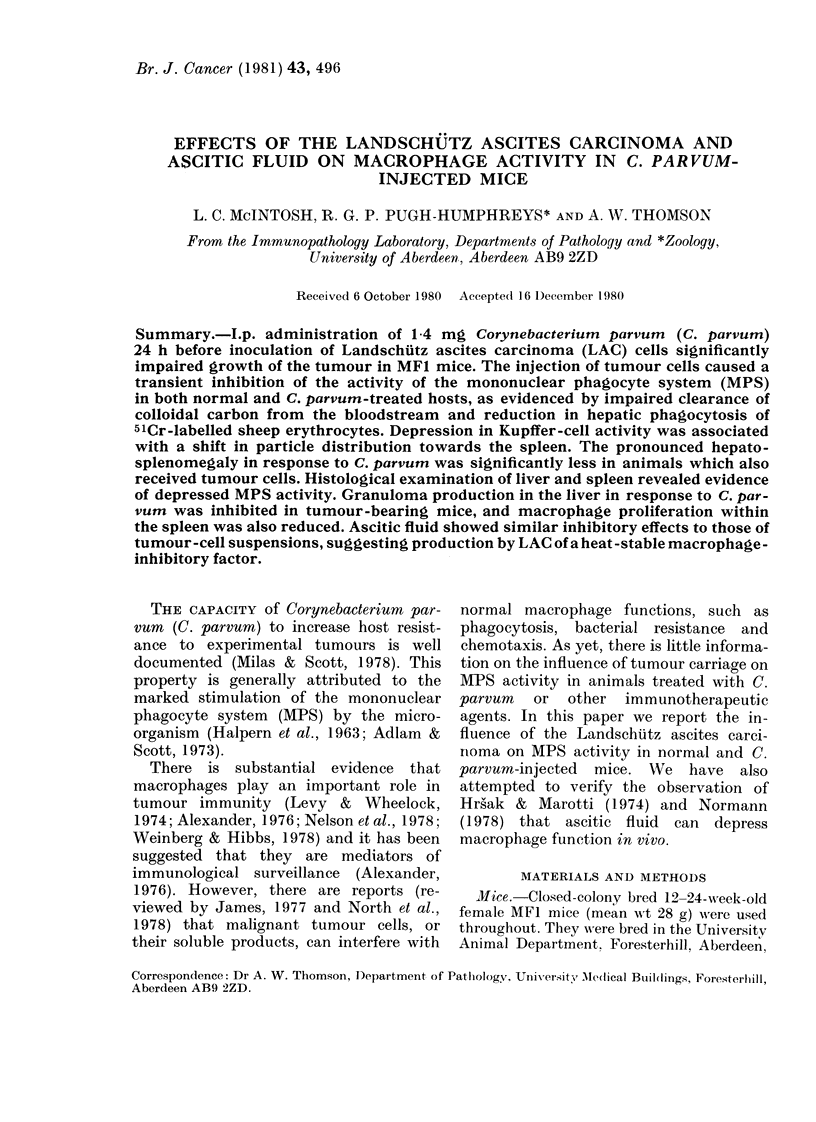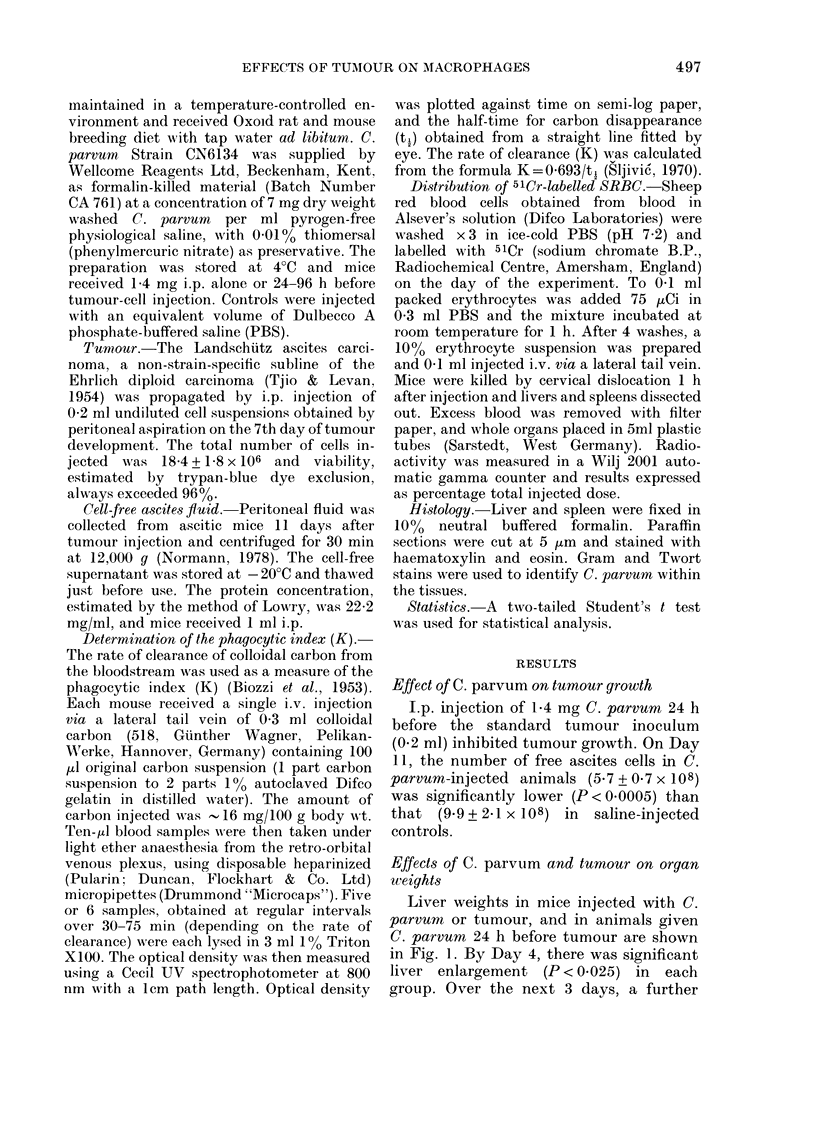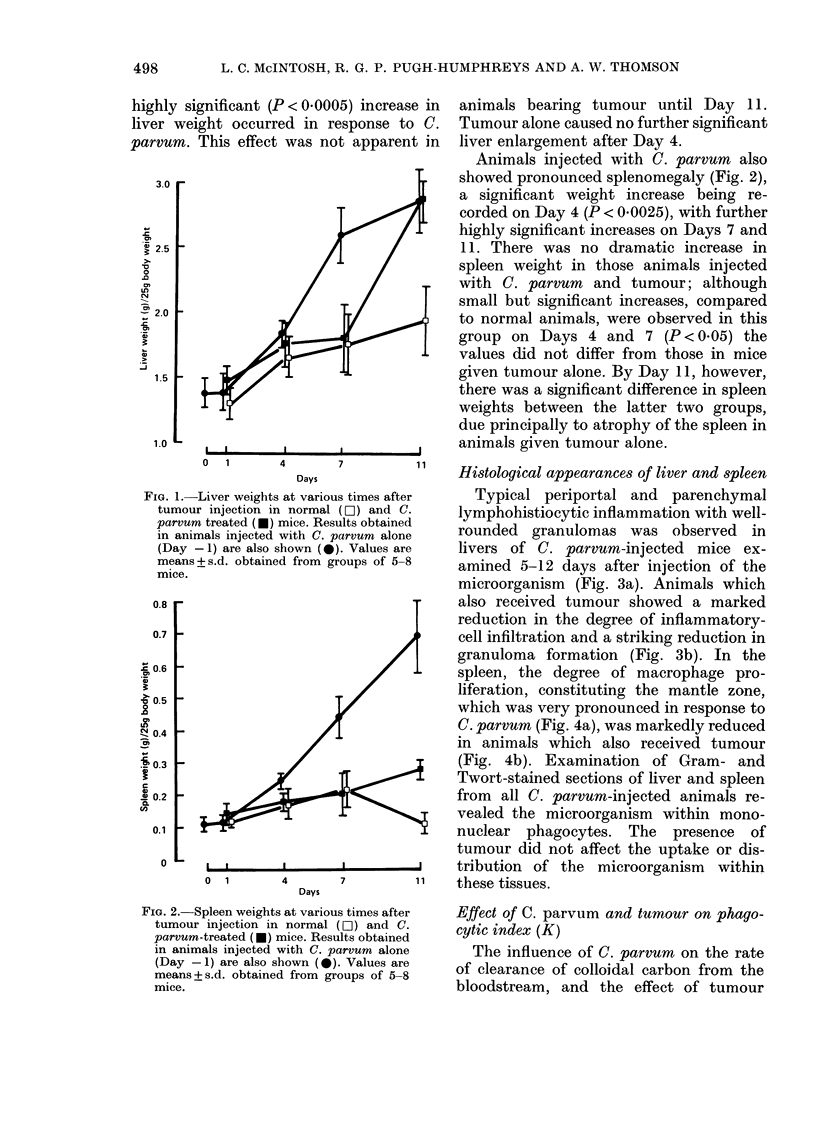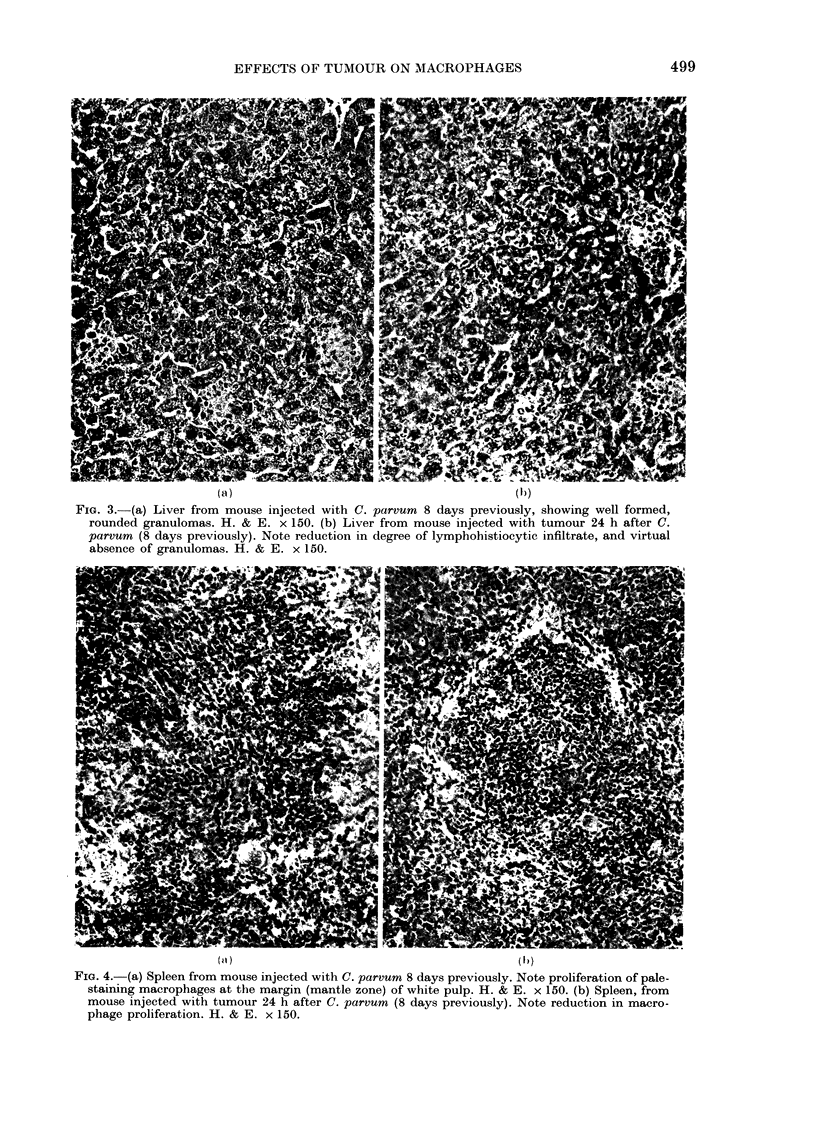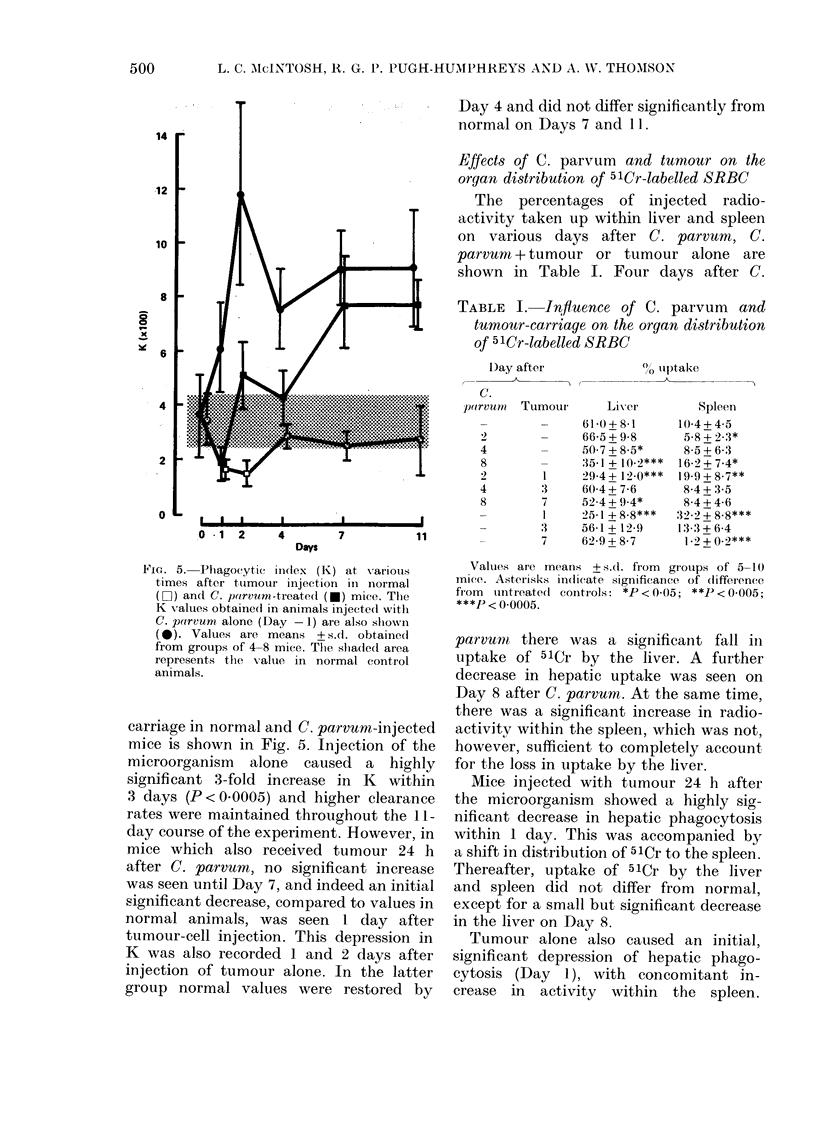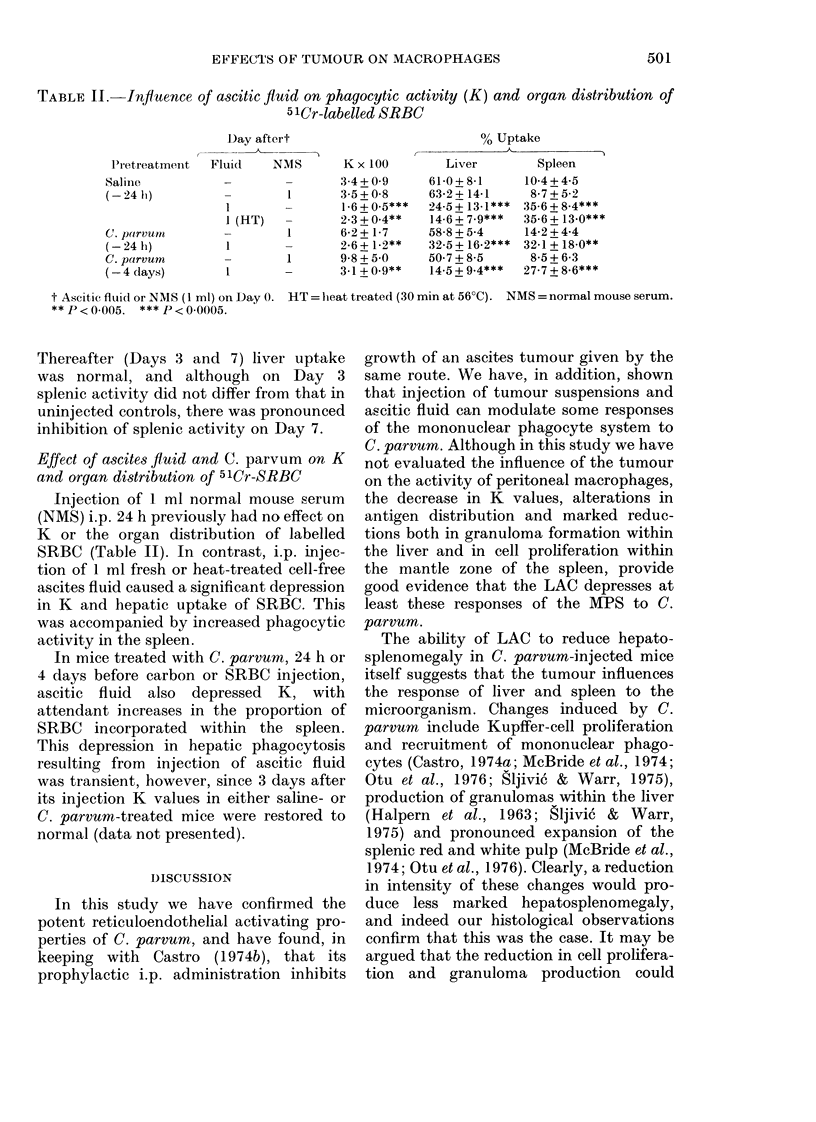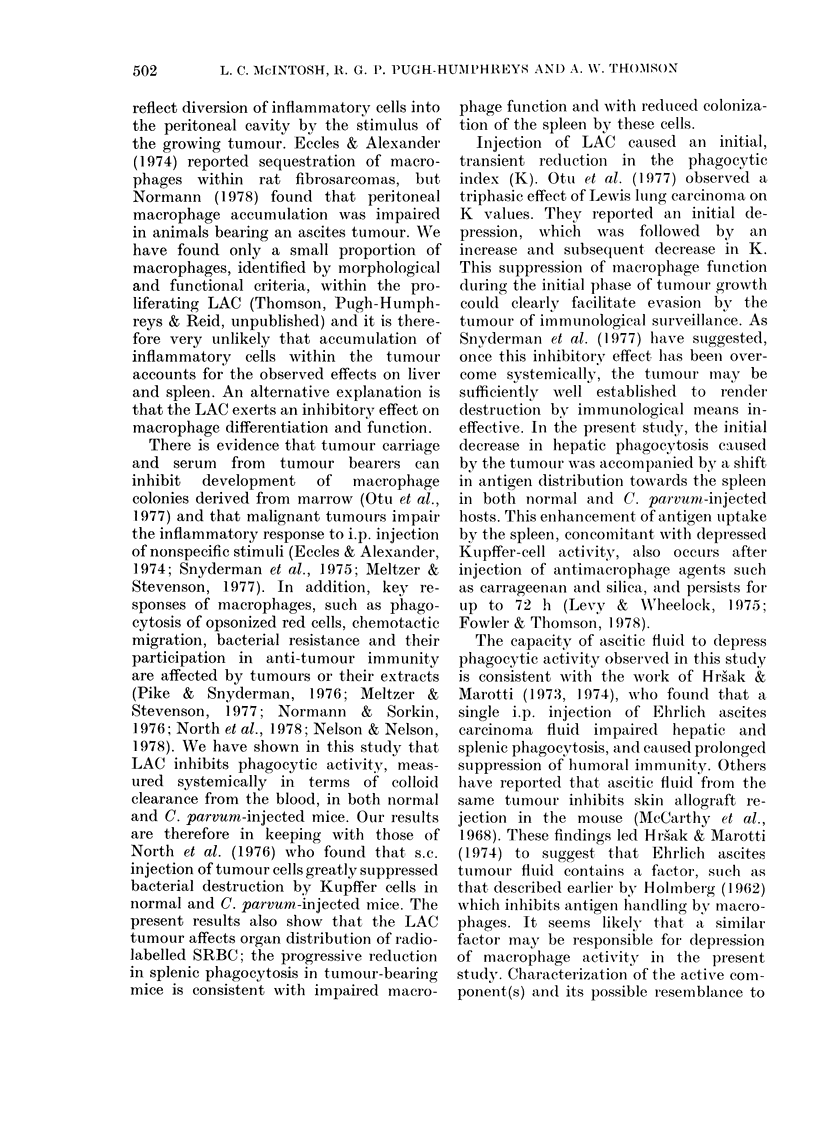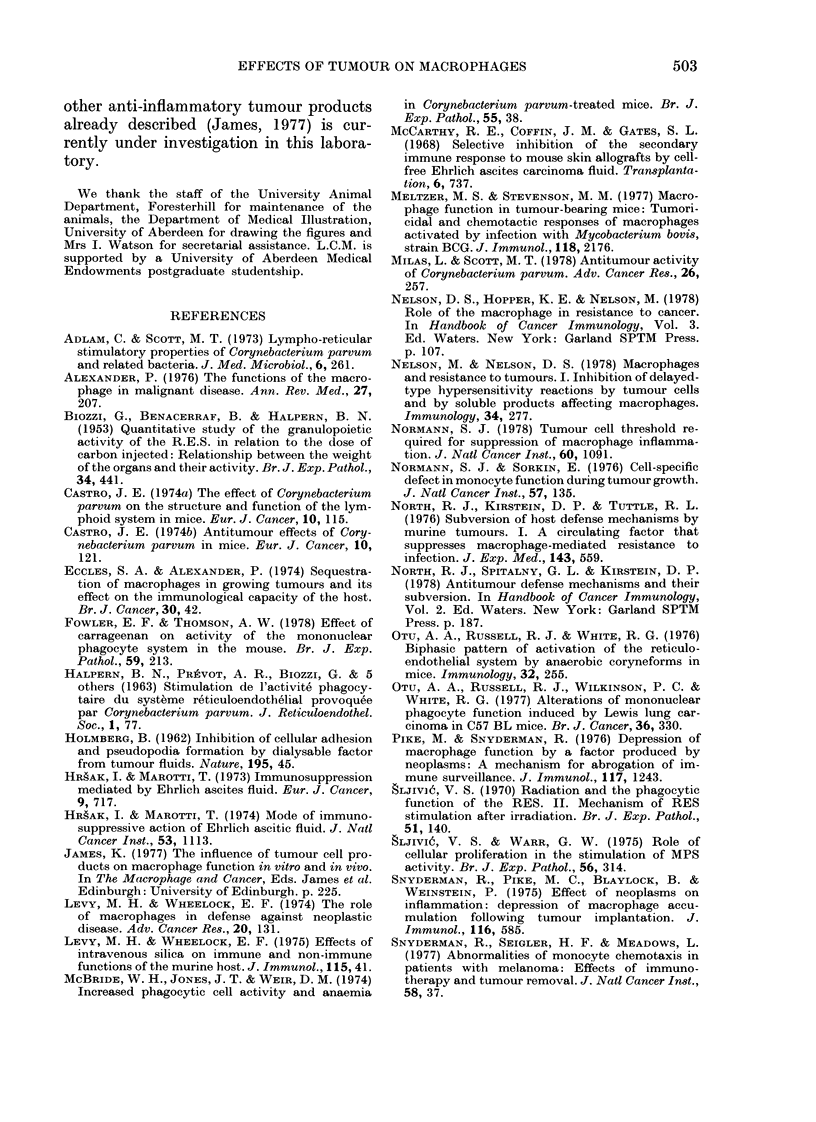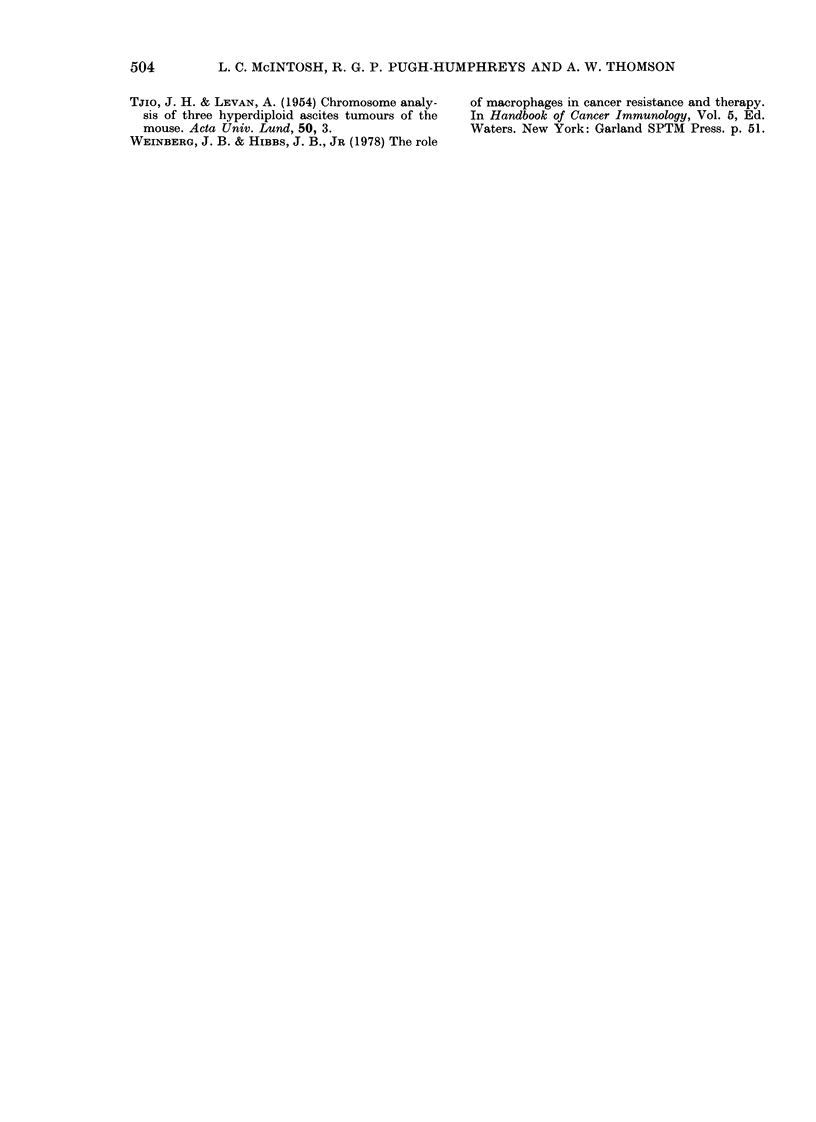# Effects of the Landschütz ascites carcinoma and ascitic fluid on macrophage activity in C. parvum-injected mice.

**DOI:** 10.1038/bjc.1981.72

**Published:** 1981-04

**Authors:** L. C. McIntosh, R. G. Pugh-Humphreys, A. W. Thomson

## Abstract

**Images:**


*Br. J. Cancer* (1981) **43**, 496

### EFFECTS OF THE LANDSCHÜTZ ASCITES CARCINOMA AND ASCITIC FLUID ON MACROPHAGE ACTIVITY IN *C. PARVUM*-INJECTED MICE

L. C. McINTOSH, R. G. P. PUGH-HUMPHREYS* AND A. W. THOMSON

*From the Immunopathology Laboratory, Departments of Pathology and *Zoology, University of Aberdeen, Aberdeen* AB9 2ZD

Received 6 October 1980 Accepted 16 December 1980

**Summary.—I.p. administration of 1·4 mg *Corynebacterium parvum* (*C. parvum*) 24 h before inoculation of Landschütz ascites carcinoma (LAC) cells significantly impaired growth of the tumour in MF1 mice. The injection of tumour cells caused a transient inhibition of the activity of the mononuclear phagocyte system (MPS) in both normal and *C. parvum*-treated hosts, as evidenced by impaired clearance of colloidal carbon from the bloodstream and reduction in hepatic phagocytosis of $^{51}Cr$-labelled sheep erythrocytes. Depression in Kupffer-cell activity was associated with a shift in particle distribution towards the spleen. The pronounced hepatosplenomegaly in response to *C. parvum* was significantly less in animals which also received tumour cells. Histological examination of liver and spleen revealed evidence of depressed MPS activity. Granuloma production in the liver in response to *C. parvum* was inhibited in tumour-bearing mice, and macrophage proliferation within the spleen was also reduced. Ascitic fluid showed similar inhibitory effects to those of tumour-cell suspensions, suggesting production by LAC of a heat-stable macrophage-inhibitory factor.**

THE CAPACITY of *Corynebacterium parvum* (*C. parvum*) to increase host resistance to experimental tumours is well documented (Milas & Scott, 1978). This property is generally attributed to the marked stimulation of the mononuclear phagocyte system (MPS) by the micro-organism (Halpern *et al.*, 1963; Adlam & Scott, 1973).

There is substantial evidence that macrophages play an important role in tumour immunity (Levy & Wheelock, 1974; Alexander, 1976; Nelson *et al.*, 1978; Weinberg & Hibbs, 1978) and it has been suggested that they are mediators of immunological surveillance (Alexander, 1976). However, there are reports (reviewed by James, 1977 and North *et al.*, 1978) that malignant tumour cells, or their soluble products, can interfere with normal macrophage functions, such as phagocytosis, bacterial resistance and chemotaxis. As yet, there is little information on the influence of tumour carriage on MPS activity in animals treated with *C. parvum* or other immunotherapeutic agents. In this paper we report the influence of the Landschütz ascites carcinoma on MPS activity in normal and *C. parvum*-injected mice. We have also attempted to verify the observation of Hršak & Marotti (1974) and Normann (1978) that ascitic fluid can depress macrophage function *in vivo*.

### MATERIALS AND METHODS

*Mice.*—Closed-colony bred 12–24-week-old female MF1 mice (mean wt 28 g) were used throughout. They were bred in the University Animal Department, Foresterhill, Aberdeen,

Correspondence: Dr A. W. Thomson, Department of Pathology, University Medical Buildings, Foresterhill, Aberdeen AB9 2ZD.

maintained in a temperature-controlled environment and received Oxoid rat and mouse breeding diet with tap water *ad libitum*. *C. parvum* Strain CN6134 was supplied by Wellcome Reagents Ltd, Beckenham, Kent, as formalin-killed material (Batch Number CA 761) at a concentration of 7 mg dry weight washed *C. parvum* per ml pyrogen-free physiological saline, with 0·01% thiomersal (phenylmercuric nitrate) as preservative. The preparation was stored at 4°C and mice received 1·4 mg i.p. alone or 24–96 h before tumour-cell injection. Controls were injected with an equivalent volume of Dulbecco A phosphate-buffered saline (PBS).

*Tumour.*—The Landschütz ascites carcinoma, a non-strain-specific subline of the Ehrlich diploid carcinoma (Tjio & Levan, 1954) was propagated by i.p. injection of 0·2 ml undiluted cell suspensions obtained by peritoneal aspiration on the 7th day of tumour development. The total number of cells injected was $18{\cdot}4 \pm 1{\cdot}8 \times 10^6$ and viability, estimated by trypan-blue dye exclusion, always exceeded 96%.

*Cell-free ascites fluid.*—Peritoneal fluid was collected from ascitic mice 11 days after tumour injection and centrifuged for 30 min at 12,000 *g* (Normann, 1978). The cell-free supernatant was stored at −20°C and thawed just before use. The protein concentration, estimated by the method of Lowry, was 22·2 mg/ml, and mice received 1 ml i.p.

*Determination of the phagocytic index (K).*—The rate of clearance of colloidal carbon from the bloodstream was used as a measure of the phagocytic index (K) (Biozzi *et al.*, 1953). Each mouse received a single i.v. injection *via* a lateral tail vein of 0·3 ml colloidal carbon (518, Günther Wagner, Pelikan-Werke, Hannover, Germany) containing 100 μl original carbon suspension (1 part carbon suspension to 2 parts 1% autoclaved Difco gelatin in distilled water). The amount of carbon injected was ~16 mg/100 g body wt. Ten-μl blood samples were then taken under light ether anaesthesia from the retro-orbital venous plexus, using disposable heparinized (Pularin; Duncan, Flockhart & Co. Ltd) micropipettes (Drummond "Microcaps"). Five or 6 samples, obtained at regular intervals over 30–75 min (depending on the rate of clearance) were each lysed in 3 ml 1% Triton X100. The optical density was then measured using a Cecil UV spectrophotometer at 800 nm with a 1cm path length. Optical density was plotted against time on semi-log paper, and the half-time for carbon disappearance ($t_{\frac{1}{2}}$) obtained from a straight line fitted by eye. The rate of clearance (K) was calculated from the formula $K = 0{\cdot}693/t_{\frac{1}{2}}$ (Šljivić, 1970).

*Distribution of $^{51}Cr$-labelled SRBC.*—Sheep red blood cells obtained from blood in Alsever's solution (Difco Laboratories) were washed ×3 in ice-cold PBS (pH 7·2) and labelled with $^{51}$Cr (sodium chromate B.P., Radiochemical Centre, Amersham, England) on the day of the experiment. To 0·1 ml packed erythrocytes was added 75 μCi in 0·3 ml PBS and the mixture incubated at room temperature for 1 h. After 4 washes, a 10% erythrocyte suspension was prepared and 0·1 ml injected i.v. *via* a lateral tail vein. Mice were killed by cervical dislocation 1 h after injection and livers and spleens dissected out. Excess blood was removed with filter paper, and whole organs placed in 5ml plastic tubes (Sarstedt, West Germany). Radioactivity was measured in a Wilj 2001 automatic gamma counter and results expressed as percentage total injected dose.

*Histology.*—Liver and spleen were fixed in 10% neutral buffered formalin. Paraffin sections were cut at 5 μm and stained with haematoxylin and eosin. Gram and Twort stains were used to identify *C. parvum* within the tissues.

*Statistics.*—A two-tailed Student's *t* test was used for statistical analysis.

### RESULTS

### *Effect of* C. parvum *on tumour growth*

I.p. injection of 1·4 mg *C. parvum* 24 h before the standard tumour inoculum (0·2 ml) inhibited tumour growth. On Day 11, the number of free ascites cells in *C. parvum*-injected animals ($5{\cdot}7 \pm 0{\cdot}7 \times 10^8$) was significantly lower ($P < 0{\cdot}0005$) than that ($9{\cdot}9 \pm 2{\cdot}1 \times 10^8$) in saline-injected controls.

### *Effects of* C. parvum *and tumour on organ weights*

Liver weights in mice injected with *C. parvum* or tumour, and in animals given *C. parvum* 24 h before tumour are shown in Fig. 1. By Day 4, there was significant liver enlargement ($P < 0{\cdot}025$) in each group. Over the next 3 days, a further highly significant ($P < 0{\cdot}0005$) increase in liver weight occurred in response to *C. parvum*. This effect was not apparent in

FIG. 1.—Liver weights at various times after tumour injection in normal (□) and *C. parvum* treated (■) mice. Results obtained in animals injected with *C. parvum* alone (Day −1) are also shown (●). Values are means ± s.d. obtained from groups of 5–8 mice.

FIG. 2.—Spleen weights at various times after tumour injection in normal (□) and *C. parvum*-treated (■) mice. Results obtained in animals injected with *C. parvum* alone (Day −1) are also shown (●). Values are means ± s.d. obtained from groups of 5–8 mice.

animals bearing tumour until Day 11. Tumour alone caused no further significant liver enlargement after Day 4.

Animals injected with *C. parvum* also showed pronounced splenomegaly (Fig. 2), a significant weight increase being recorded on Day 4 ($P < 0{\cdot}0025$), with further highly significant increases on Days 7 and 11. There was no dramatic increase in spleen weight in those animals injected with *C. parvum* and tumour; although small but significant increases, compared to normal animals, were observed in this group on Days 4 and 7 ($P < 0{\cdot}05$) the values did not differ from those in mice given tumour alone. By Day 11, however, there was a significant difference in spleen weights between the latter two groups, due principally to atrophy of the spleen in animals given tumour alone.

*Histological appearances of liver and spleen*

Typical periportal and parenchymal lymphohistiocytic inflammation with well-rounded granulomas was observed in livers of *C. parvum*-injected mice examined 5–12 days after injection of the microorganism (Fig. 3a). Animals which also received tumour showed a marked reduction in the degree of inflammatory-cell infiltration and a striking reduction in granuloma formation (Fig. 3b). In the spleen, the degree of macrophage proliferation, constituting the mantle zone, which was very pronounced in response to *C. parvum* (Fig. 4a), was markedly reduced in animals which also received tumour (Fig. 4b). Examination of Gram- and Twort-stained sections of liver and spleen from all *C. parvum*-injected animals revealed the microorganism within mononuclear phagocytes. The presence of tumour did not affect the uptake or distribution of the microorganism within these tissues.

*Effect of* C. parvum *and tumour on phagocytic index* (*K*)

The influence of *C. parvum* on the rate of clearance of colloidal carbon from the bloodstream, and the effect of tumour

(a) (b)

FIG. 3.—(a) Liver from mouse injected with *C. parvum* 8 days previously, showing well formed, rounded granulomas. H. & E. × 150. (b) Liver from mouse injected with tumour 24 h after *C. parvum* (8 days previously). Note reduction in degree of lymphohistiocytic infiltrate, and virtual absence of granulomas. H. & E. × 150.

(a) (b)

FIG. 4.—(a) Spleen from mouse injected with *C. parvum* 8 days previously. Note proliferation of pale-staining macrophages at the margin (mantle zone) of white pulp. H. & E. × 150. (b) Spleen, from mouse injected with tumour 24 h after *C. parvum* (8 days previously). Note reduction in macrophage proliferation. H. & E. × 150.

FIG. 5.—Phagocytic index (K) at various times after tumour injection in normal (□) and *C. parvum*-treated (■) mice. The K values obtained in animals injected with *C. parvum* alone (Day −1) are also shown (●). Values are means ±s.d. obtained from groups of 4–8 mice. The shaded area represents the value in normal control animals.

carriage in normal and *C. parvum*-injected mice is shown in Fig. 5. Injection of the microorganism alone caused a highly significant 3-fold increase in K within 3 days ($P<0.0005$) and higher clearance rates were maintained throughout the 11-day course of the experiment. However, in mice which also received tumour 24 h after *C. parvum*, no significant increase was seen until Day 7, and indeed an initial significant decrease, compared to values in normal animals, was seen 1 day after tumour-cell injection. This depression in K was also recorded 1 and 2 days after injection of tumour alone. In the latter group normal values were restored by Day 4 and did not differ significantly from normal on Days 7 and 11.

*Effects of* C. parvum *and tumour on the organ distribution of* $^{51}$*Cr-labelled SRBC*

The percentages of injected radioactivity taken up within liver and spleen on various days after *C. parvum*, *C. parvum*+tumour or tumour alone are shown in Table I. Four days after *C.*

TABLE I.—*Influence of* C. parvum *and tumour-carriage on the organ distribution of* $^{51}$*Cr-labelled SRBC*

| Day after | | % uptake | |
|---|---|---|---|
| *C. parvum* | Tumour | Liver | Spleen |
| – | – | 61·0±8·1 | 10·4±4·5 |
| 2 | – | 66·5±9·8 | 5·8±2·3* |
| 4 | – | 50·7±8·5* | 8·5±6·3 |
| 8 | – | 35·1±10·2*** | 16·2±7·4* |
| 2 | 1 | 29·4±12·0*** | 19·9±8·7** |
| 4 | 3 | 60·4±7·6 | 8·4±3·5 |
| 8 | 7 | 52·4±9·4* | 8·4±4·6 |
| – | 1 | 25·1±8·8*** | 32·2±8·8*** |
| – | 3 | 56·1±12·9 | 13·3±6·4 |
| – | 7 | 62·9±8·7 | 1·2±0·2*** |

Values are means ±s.d. from groups of 5–10 mice. Asterisks indicate significance of difference from untreated controls: *$P<0.05$; **$P<0.005$; ***$P<0.0005$.

*parvum* there was a significant fall in uptake of $^{51}$Cr by the liver. A further decrease in hepatic uptake was seen on Day 8 after *C. parvum*. At the same time, there was a significant increase in radioactivity within the spleen, which was not, however, sufficient to completely account for the loss in uptake by the liver.

Mice injected with tumour 24 h after the microorganism showed a highly significant decrease in hepatic phagocytosis within 1 day. This was accompanied by a shift in distribution of $^{51}$Cr to the spleen. Thereafter, uptake of $^{51}$Cr by the liver and spleen did not differ from normal, except for a small but significant decrease in the liver on Day 8.

Tumour alone also caused an initial, significant depression of hepatic phagocytosis (Day 1), with concomitant increase in activity within the spleen.

TABLE II.—*Influence of ascitic fluid on phagocytic activity (K) and organ distribution of $^{51}$Cr-labelled SRBC*

| | Day after† | | | % Uptake | |
|---|---|---|---|---|---|
| Pretreatment | Fluid | NMS | K × 100 | Liver | Spleen |
| Saline | – | – | 3·4 ± 0·9 | 61·0 ± 8·1 | 10·4 ± 4·5 |
| (−24 h) | – | 1 | 3·5 ± 0·8 | 63·2 ± 14·1 | 8·7 ± 5·2 |
| | 1 | – | 1·6 ± 0·5*** | 24·5 ± 13·1*** | 35·6 ± 8·4*** |
| | 1 (HT) | – | 2·3 ± 0·4** | 14·6 ± 7·9*** | 35·6 ± 13·0*** |
| *C. parvum* | – | 1 | 6·2 ± 1·7 | 58·8 ± 5·4 | 14·2 ± 4·4 |
| (−24 h) | 1 | – | 2·6 ± 1·2** | 32·5 ± 16·2*** | 32·1 ± 18·0** |
| *C. parvum* | – | 1 | 9·8 ± 5·0 | 50·7 ± 8·5 | 8·5 ± 6·3 |
| (−4 days) | 1 | – | 3·1 ± 0·9** | 14·5 ± 9·4*** | 27·7 ± 8·6*** |

† Ascitic fluid or NMS (1 ml) on Day 0. HT = heat treated (30 min at 56°C). NMS = normal mouse serum.

** $P < 0·005$. *** $P < 0·0005$.

Thereafter (Days 3 and 7) liver uptake was normal, and although on Day 3 splenic activity did not differ from that in uninjected controls, there was pronounced inhibition of splenic activity on Day 7.

*Effect of ascites fluid and* C. parvum *on K and organ distribution of $^{51}$Cr-SRBC*

Injection of 1 ml normal mouse serum (NMS) i.p. 24 h previously had no effect on K or the organ distribution of labelled SRBC (Table II). In contrast, i.p. injection of 1 ml fresh or heat-treated cell-free ascites fluid caused a significant depression in K and hepatic uptake of SRBC. This was accompanied by increased phagocytic activity in the spleen.

In mice treated with *C. parvum*, 24 h or 4 days before carbon or SRBC injection, ascitic fluid also depressed K, with attendant increases in the proportion of SRBC incorporated within the spleen. This depression in hepatic phagocytosis resulting from injection of ascitic fluid was transient, however, since 3 days after its injection K values in either saline- or *C. parvum*-treated mice were restored to normal (data not presented).

### DISCUSSION

In this study we have confirmed the potent reticuloendothelial activating properties of *C. parvum*, and have found, in keeping with Castro (1974*b*), that its prophylactic i.p. administration inhibits growth of an ascites tumour given by the same route. We have, in addition, shown that injection of tumour suspensions and ascitic fluid can modulate some responses of the mononuclear phagocyte system to *C. parvum*. Although in this study we have not evaluated the influence of the tumour on the activity of peritoneal macrophages, the decrease in K values, alterations in antigen distribution and marked reductions both in granuloma formation within the liver and in cell proliferation within the mantle zone of the spleen, provide good evidence that the LAC depresses at least these responses of the MPS to *C. parvum*.

The ability of LAC to reduce hepatosplenomegaly in *C. parvum*-injected mice itself suggests that the tumour influences the response of liver and spleen to the microorganism. Changes induced by *C. parvum* include Kupffer-cell proliferation and recruitment of mononuclear phagocytes (Castro, 1974*a*; McBride *et al.*, 1974; Otu *et al.*, 1976; Šljivić & Warr, 1975), production of granulomas within the liver (Halpern *et al.*, 1963; Šljivić & Warr, 1975) and pronounced expansion of the splenic red and white pulp (McBride *et al.*, 1974; Otu *et al.*, 1976). Clearly, a reduction in intensity of these changes would produce less marked hepatosplenomegaly, and indeed our histological observations confirm that this was the case. It may be argued that the reduction in cell proliferation and granuloma production could reflect diversion of inflammatory cells into the peritoneal cavity by the stimulus of the growing tumour. Eccles & Alexander (1974) reported sequestration of macrophages within rat fibrosarcomas, but Normann (1978) found that peritoneal macrophage accumulation was impaired in animals bearing an ascites tumour. We have found only a small proportion of macrophages, identified by morphological and functional criteria, within the proliferating LAC (Thomson, Pugh-Humphreys & Reid, unpublished) and it is therefore very unlikely that accumulation of inflammatory cells within the tumour accounts for the observed effects on liver and spleen. An alternative explanation is that the LAC exerts an inhibitory effect on macrophage differentiation and function.

There is evidence that tumour carriage and serum from tumour bearers can inhibit development of macrophage colonies derived from marrow (Otu *et al.*, 1977) and that malignant tumours impair the inflammatory response to i.p. injection of nonspecific stimuli (Eccles & Alexander, 1974; Snyderman *et al.*, 1975; Meltzer & Stevenson, 1977). In addition, key responses of macrophages, such as phagocytosis of opsonized red cells, chemotactic migration, bacterial resistance and their participation in anti-tumour immunity are affected by tumours or their extracts (Pike & Snyderman, 1976; Meltzer & Stevenson, 1977; Normann & Sorkin, 1976; North *et al.*, 1978; Nelson & Nelson, 1978). We have shown in this study that LAC inhibits phagocytic activity, measured systemically in terms of colloid clearance from the blood, in both normal and *C. parvum*-injected mice. Our results are therefore in keeping with those of North *et al.* (1976) who found that s.c. injection of tumour cells greatly suppressed bacterial destruction by Kupffer cells in normal and *C. parvum*-injected mice. The present results also show that the LAC tumour affects organ distribution of radiolabelled SRBC; the progressive reduction in splenic phagocytosis in tumour-bearing mice is consistent with impaired macrophage function and with reduced colonization of the spleen by these cells.

Injection of LAC caused an initial, transient reduction in the phagocytic index (K). Otu *et al.* (1977) observed a triphasic effect of Lewis lung carcinoma on K values. They reported an initial depression, which was followed by an increase and subsequent decrease in K. This suppression of macrophage function during the initial phase of tumour growth could clearly facilitate evasion by the tumour of immunological surveillance. As Snyderman *et al.* (1977) have suggested, once this inhibitory effect has been overcome systemically, the tumour may be sufficiently well established to render destruction by immunological means ineffective. In the present study, the initial decrease in hepatic phagocytosis caused by the tumour was accompanied by a shift in antigen distribution towards the spleen in both normal and *C. parvum*-injected hosts. This enhancement of antigen uptake by the spleen, concomitant with depressed Kupffer-cell activity, also occurs after injection of antimacrophage agents such as carrageenan and silica, and persists for up to 72 h (Levy & Wheelock, 1975; Fowler & Thomson, 1978).

The capacity of ascitic fluid to depress phagocytic activity observed in this study is consistent with the work of Hršak & Marotti (1973, 1974), who found that a single i.p. injection of Ehrlich ascites carcinoma fluid impaired hepatic and splenic phagocytosis, and caused prolonged suppression of humoral immunity. Others have reported that ascitic fluid from the same tumour inhibits skin allograft rejection in the mouse (McCarthy *et al.*, 1968). These findings led Hršak & Marotti (1974) to suggest that Ehrlich ascites tumour fluid contains a factor, such as that described earlier by Holmberg (1962) which inhibits antigen handling by macrophages. It seems likely that a similar factor may be responsible for depression of macrophage activity in the present study. Characterization of the active component(s) and its possible resemblance to other anti-inflammatory tumour products already described (James, 1977) is currently under investigation in this laboratory.

We thank the staff of the University Animal Department, Foresterhill for maintenance of the animals, the Department of Medical Illustration, University of Aberdeen for drawing the figures and Mrs I. Watson for secretarial assistance. L.C.M. is supported by a University of Aberdeen Medical Endowments postgraduate studentship.

### REFERENCES

ADLAM, C. & SCOTT, M. T. (1973) Lympho-reticular stimulatory properties of *Corynebacterium parvum* and related bacteria. *J. Med. Microbiol.*, **6**, 261.

ALEXANDER, P. (1976) The functions of the macrophage in malignant disease. *Ann. Rev. Med.*, **27**, 207.

BIOZZI, G., BENACERRAF, B. & HALPERN, B. N. (1953) Quantitative study of the granulopoietic activity of the R.E.S. in relation to the dose of carbon injected: Relationship between the weight of the organs and their activity. *Br. J. Exp. Pathol.*, **34**, 441.

CASTRO, J. E. (1974*a*) The effect of *Corynebacterium parvum* on the structure and function of the lymphoid system in mice. *Eur. J. Cancer*, **10**, 115.

CASTRO, J. E. (1974*b*) Antitumour effects of *Corynebacterium parvum* in mice. *Eur. J. Cancer*, **10**, 121.

ECCLES, S. A. & ALEXANDER, P. (1974) Sequestration of macrophages in growing tumours and its effect on the immunological capacity of the host. *Br. J. Cancer*, **30**, 42.

FOWLER, E. F. & THOMSON, A. W. (1978) Effect of carrageenan on activity of the mononuclear phagocyte system in the mouse. *Br. J. Exp. Pathol.*, **59**, 213.

HALPERN, B. N., PRÉVOT, A. R., BIOZZI, G. & 5 others (1963) Stimulation de l'activité phagocytaire du système réticuloendothélial provoquée par *Corynebacterium parvum*. *J. Reticuloendothel. Soc.*, **1**, 77.

HOLMBERG, B. (1962) Inhibition of cellular adhesion and pseudopodia formation by dialysable factor from tumour fluids. *Nature*, **195**, 45.

HRŠAK, I. & MAROTTI, T. (1973) Immunosuppression mediated by Ehrlich ascites fluid. *Eur. J. Cancer*, **9**, 717.

HRŠAK, I. & MAROTTI, T. (1974) Mode of immunosuppressive action of Ehrlich ascitic fluid. *J. Natl Cancer Inst.*, **53**, 1113.

JAMES, K. (1977) The influence of tumour cell products on macrophage function *in vitro* and *in vivo*. In *The Macrophage and Cancer*, Eds. James *et al.* Edinburgh: University of Edinburgh. p. 225.

LEVY, M. H. & WHEELOCK, E. F. (1974) The role of macrophages in defense against neoplastic disease. *Adv. Cancer Res.*, **20**, 131.

LEVY, M. H. & WHEELOCK, E. F. (1975) Effects of intravenous silica on immune and non-immune functions of the murine host. *J. Immunol.*, **115**, 41.

MCBRIDE, W. H., JONES, J. T. & WEIR, D. M. (1974) Increased phagocytic cell activity and anaemia in *Corynebacterium parvum*-treated mice. *Br. J. Exp. Pathol.*, **55**, 38.

MCCARTHY, R. E., COFFIN, J. M. & GATES, S. L. (1968) Selective inhibition of the secondary immune response to mouse skin allografts by cell-free Ehrlich ascites carcinoma fluid. *Transplantation*, **6**, 737.

MELTZER, M. S. & STEVENSON, M. M. (1977) Macrophage function in tumour-bearing mice: Tumoricidal and chemotactic responses of macrophages activated by infection with *Mycobacterium bovis*, strain BCG. *J. Immunol.*, **118**, 2176.

MILAS, L. & SCOTT, M. T. (1978) Antitumour activity of *Corynebacterium parvum*. *Adv. Cancer Res.*, **26**, 257.

NELSON, D. S., HOPPER, K. E. & NELSON, M. (1978) Role of the macrophage in resistance to cancer. In *Handbook of Cancer Immunology*, Vol. 3. Ed. Waters. New York: Garland SPTM Press. p. 107.

NELSON, M. & NELSON, D. S. (1978) Macrophages and resistance to tumours. I. Inhibition of delayed-type hypersensitivity reactions by tumour cells and by soluble products affecting macrophages. *Immunology*, **34**, 277.

NORMANN, S. J. (1978) Tumour cell threshold required for suppression of macrophage inflammation. *J. Natl Cancer Inst.*, **60**, 1091.

NORMANN, S. J. & SORKIN, E. (1976) Cell-specific defect in monocyte function during tumour growth. *J. Natl Cancer Inst.*, **57**, 135.

NORTH, R. J., KIRSTEIN, D. P. & TUTTLE, R. L. (1976) Subversion of host defense mechanisms by murine tumours. I. A circulating factor that suppresses macrophage-mediated resistance to infection. *J. Exp. Med.*, **143**, 559.

NORTH, R. J., SPITALNY, G. L. & KIRSTEIN, D. P. (1978) Antitumour defense mechanisms and their subversion. In *Handbook of Cancer Immunology*, Vol. 2. Ed. Waters. New York: Garland SPTM Press. p. 187.

OTU, A. A., RUSSELL, R. J. & WHITE, R. G. (1976) Biphasic pattern of activation of the reticuloendothelial system by anaerobic coryneforms in mice. *Immunology*, **32**, 255.

OTU, A. A., RUSSELL, R. J., WILKINSON, P. C. & WHITE, R. G. (1977) Alterations of mononuclear phagocyte function induced by Lewis lung carcinoma in C57 BL mice. *Br. J. Cancer*, **36**, 330.

PIKE, M. & SNYDERMAN, R. (1976) Depression of macrophage function by a factor produced by neoplasms: A mechanism for abrogation of immune surveillance. *J. Immunol.*, **117**, 1243.

ŠLJIVIĆ, V. S. (1970) Radiation and the phagocytic function of the RES. II. Mechanism of RES stimulation after irradiation. *Br. J. Exp. Pathol.*, **51**, 140.

ŠLJIVIĆ, V. S. & WARR, G. W. (1975) Role of cellular proliferation in the stimulation of MPS activity. *Br. J. Exp. Pathol.*, **56**, 314.

SNYDERMAN, R., PIKE, M. C., BLAYLOCK, B. & WEINSTEIN, P. (1975) Effect of neoplasms on inflammation: depression of macrophage accumulation following tumour implantation. *J. Immunol.*, **116**, 585.

SNYDERMAN, R., SEIGLER, H. F. & MEADOWS, L. (1977) Abnormalities of monocyte chemotaxis in patients with melanoma: Effects of immunotherapy and tumour removal. *J. Natl Cancer Inst.*, **58**, 37.

TJIO, J. H. & LEVAN, A. (1954) Chromosome analysis of three hyperdiploid ascites tumours of the mouse. *Acta Univ. Lund*, **50**, 3.
WEINBERG, J. B. & HIBBS, J. B., JR (1978) The role of macrophages in cancer resistance and therapy. In *Handbook of Cancer Immunology*, Vol. 5, Ed. Waters. New York: Garland SPTM Press. p. 51.